# Cell death in uveitis: pyroptosis, ferroptosis, apoptosis, and beyond

**DOI:** 10.3389/fimmu.2026.1751523

**Published:** 2026-05-05

**Authors:** Jianxin Liu, Liming Mao, Guoqing Chen, Liang Zou, Yi Shi, Lingxi Jiang, Jiayu Meng

**Affiliations:** 1 Genetic Diseases Key Laboratory of Sichuan Province, Sichuan Provincial People’s Hospital, School of Medicine, University of Electronic Science and Technology of China, Chengdu, China; 2 School of Food and Biological Engineering, Chengdu University, Chengdu, China; 3 Department of Immunology, School of Medicine, Nantong University, Nantong, Jiangsu, China; 4 Department of Ophthalmology, Sichuan Provincial People’s Hospital, University of Electronic Science and Technology of China, Chengdu, China; 5 Genetic Diseases Key Laboratory of Sichuan Province, Department of Laboratory Medicine, Sichuan Academy of Medical Sciences and Sichuan Provincial People’s Hospital, University of Electronic Science and Technology of China, Chengdu, China; 6 Research Unit for Blindness Prevention of Chinese Academy of Medical Sciences (2019RU026), Sichuan Academy of Medical Sciences and Sichuan Provincial People’s Hospital, Chengdu, China; 7 Sichuan-Chongqing Joint Key Laboratory of Pathology and Laboratory Medicine, Jinfeng Laboratory, Chongqing, China

**Keywords:** cell death, uveitis, pyroptosis, ferroptosis, apoptosis, NETosis, autophagic cell death, necroptosis

## Abstract

Uveitis is a group of diseases characterized by intraocular inflammation, closely associated with infectious, autoimmune, genetic, or environmental factors. Cell death, a critical biological process involved in development, the maintenance of homeostasis, and the pathogenesis of disease, serves as both a trigger for inflammation and a direct cause of tissue damage in uveitis. This review comprehensively summarized the roles of various forms of cell death in the pathogenesis of uveitis, providing a novel theoretical framework for advancing the understanding of its underlying mechanisms, subtype classification, prognosis evaluation, and biomarker development. Furthermore, we analyzed the efficacy and potential applications of therapeutic strategies targeting these cell death pathways, thereby laying a foundation for exploring personalized combination therapies for uveitis.

## 1 Introduction

Cell death refers to the irreversible loss of cellular structure and function caused by physiological or pathological factors. It is a critical process that plays essential roles in organismal development, homeostasis maintenance, and disease pathogenesis (1). Based on morphological features and underlying molecular mechanisms, cell death can be classified into several categories: programmed cell death (also termed regulated cell death, including apoptosis, autophagic cell death, necroptosis, pyroptosis and ferroptosis), accidental cell death (non-regulated cell death, such as necrosis), as well as other specialized forms such as neutrophil extracellular trap osis (NETosis) and parthanatos (2–5).

Aberrent cell death is closely associated with various diseases, and its study holds significant implications for understanding the essence of life processes and for advancing disease treatment, especially in the context of tumors, neurodegenerative diseases, and inflammatory conditions (6–10). Pyroptosis and necroptosis trigger immune responses by releasing damage-associated molecular patterns (DAMPs), while apoptosis eliminates excess cells such as the regression of interdigital webs during embryonic development (11–14). From a therapeutic perspective, pro-apoptotic drugs such as the B-cell lymphoma-2 (Bcl-2) inhibitor venetoclax are currently used in the treatment of leukemia, whereas inhibitors targeting NOD-like receptor family pyrin domain containing 3 (NLRP3) or gasdermin D (key mediators of pyroptosis) show promise in reducing inflammation (15–20). Furthermore, research into cell death mechanisms provides critical insights into the development of biomarkers and tumor-targeted drug delivery systems (21–24).

Uveitis is a leading cause of vision-threatening ocular inflammation worldwide, characterized by a complex pathogenesis involving immune cell infiltration, oxidative stress, and various forms of cell death (25–32). In this review, we systematically summarize recent advances in key cell death pathways implicated in uveitis pathogenesis, including pyroptosis, ferroptosis, apoptosis, NETosis, autophagic cell death, and necroptosis, and discuss their roles in disease initiation and progression (see **Table 1**). These insights provide a novel theoretical framework for understanding uveitis pathology. Furthermore, we highlight the therapeutic potential of targeting these specific cell death mechanisms, which may facilitate the development of uveitis-specific biomarkers and inform future strategies for targeted combination therapies (see **Table 2**).

**Table 1:** Characteristics and roles of cell death.

Cell death	Phenotypes	Target genes	Mechanism of response	Roles in uveitis
Pyroptosis	Membrane pores, release of intracellular contents	Inflammasomes, gasdermin family, interleukin (IL)-1β, IL-18 (36–38)	Pro-inflammatory	Amplifies the inflammatory cascades
Ferroptosis	Glutathione (GSH) depletion, lipid peroxidation, loss of mitochondrial cristae (39, 40)	Reactive oxygen species (ROS), glutathione peroxidase 4 (GPX4) (41)	Pro-inflammatory	‘Ferroptosis-inflammation’ positive feedback
Apoptosis	Cell shrinkage, chromatin condensation, apoptotic bodies (42, 43)	Caspase3/7/8/9 (44, 45)	Dual role: maintains immune privilege, but suppressed in uveitis	T cell imbalance
NETosis	DNA reticular structures (46, 47)	Peptidyl arginine deiminase 4 (PAD4), myeloperoxidase (MPO), neutrophil elastase (NE) (48)	Pro-inflammatory	Breakdown of blood-retinal barrier (BRB)
Autophagic cell death	Lysosomal degradation, autophagolysosomes (49)	Autophagy-related gene (ATG) family, beclin-1 (50, 51)	Dual role: mainly anti-inflammatory	Clears pathogens and damaged mitochondria, prevent Th17 activation
Necroptosis	Cell and organelle swelling, membrane rupture	Receptor-interacting protein kinase (RIPK)1/RIPK3/mixed lineage kinase domain-like (MLKL) (52)	Pro-inflammatory	Alternative to apoptosis

**Table 2:** Uveitis-specific biomarkers and therapeutic strategies.

Cell death	Biomarkers	Treatment strategies	Research phase
Pyroptosis	Caspase-1/4/5/11, gasdermin D, IL-1β, IL-18	MCC950/CY-09: NLRP3 (53, 54), necrosulfonamide: gasdermin D (55), anakinra/canakinumab: IL-1 (56–58)	Clinical research (58)
Ferroptosis	Iron, GPX4, ROS	Transferrin receptor (TfR) 1/divalent metal transporter (DMT) 1 inhibitors: TfR1/DMT1 (59, 60), deferoxamine/deferasirox: iron chelators (61–64), ferrostatin-1/liproxstatin-1: lipid peroxidation (65–67), selenium: GPX4 (68, 69)	Preclinical study: mouse models (70, 71)
Apoptosis	Caspase-3, Fas/FasL	Combinant agonists: FasL (72)	Basic research (73, 74)
NETosis	PAD4, MPO, NE	Paquinimod: S100A8/A9 (75, 76), PAD4 inhibitors: NETosis release (77, 78), DNase I: degrades NETosis (79, 80)	Basic research (81)
Autophagic cell death	Beclin-1, LC3-II/I, p62	Rapamycin: mTOR (82), chloroquine/hydroxychloroquine: autophagy inhibitors (83)	Preclinical study: mouse models (82)
Necroptosis	p-RIPK1/RIPK3, p-MLKL	TAT-N24: RIPK1/RIPK3 (35), Necrostatin-1: RIPK1 (84)	Early exploratory (35)

Different forms of cell death, acting through distinct molecular mechanisms, serve a double-edged role in uveitis. Pyroptosis is fundamentally an innate immune mechanism directed against intracellular pathogens; however, in the context of sterile inflammation, it can induce substantial tissue damage. Infiltrating macrophages and activated retinal microglial cells represent the principal cell types undergoing pyroptosis, thereby functioning as amplifiers of the inflammatory response. Ferroptosis and NETosis are emerging forms of cell death that have recently garnered attention in ophthalmic diseases, including uveitis. Both have been shown to promote Th17 cell activation, thereby contributing to the exacerbation of uveitis (33, 34). Apoptosis plays a critical role in the active resolution of uveitis; nevertheless, upon disruption of immune homeostasis, it may also contribute to disease progression. Autophagy, while not a form of cell death per se, regulates the various cell death pathways described above, and its impairment is typically associated with aggravated uveitis. The molecular mechanisms by which necroptosis mediates uveitis remain incompletely understood. Nonetheless, studies have demonstrated that inhibition of necroptosis-related biomarkers alleviates inflammation in animal models of uveitis, indirectly suggesting that necroptosis contributes to the pathogenesis of this condition (35).

## 2 Pyroptosis

Pyroptosis was first proposed by Cookson and Brennan in 2001 to describe a pro-inflammatory form of programmed cell death that is dependent on caspase-1 (85). Subsequent studies revealed that caspase-4, -5 and -11 could also trigger pyroptosis, thereby expanding the understanding of its underlying molecular mechanisms (86–88). Morphologically, pyroptosis is characterized by the formation of membrane pores, cellular swelling, and eventual membrane rupture, accompanied by the release of intracellular contents. The process involves the activation of gasdermin family proteins such as gasdermin D and inflammasomes like NLRP3, and the subsequent release of pro-inflammatory cytokines, including IL-1β and IL-18, which serve as key molecular indicators of pyroptosis.

**Figure 1:**
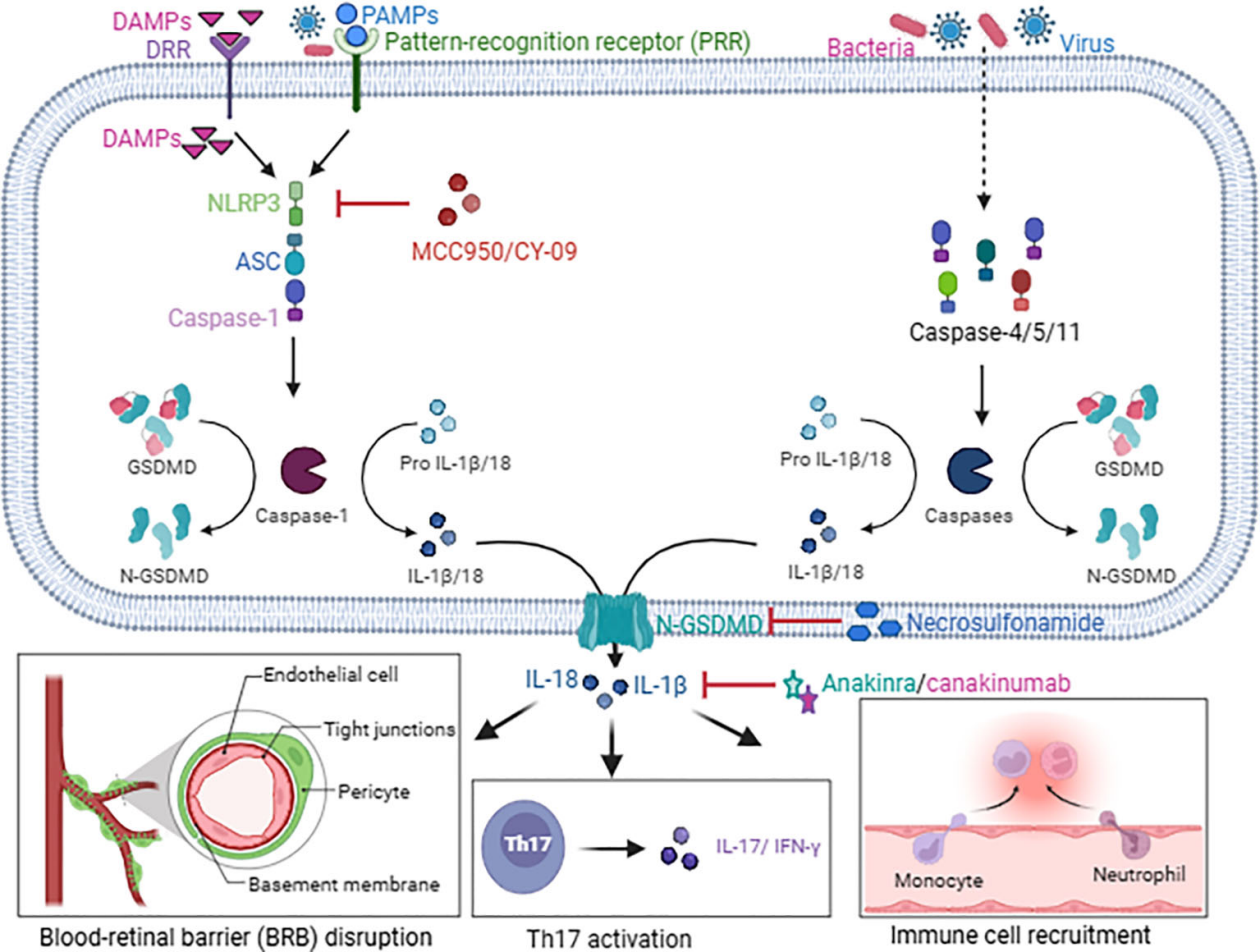
The pro-inflammatory mechanisms of pyroptosis in uveitis and related inhibitors. DAMPs activate NLRP3 either through DRR or directly, while PAMPs activate NLRP3 via PRR. This triggers the assembly of the ASC-caspase-1 complex, leading to caspase-1 activation and gasdermin D cleavage, which in turn facilitates the release of mature IL-18 and IL-1β. Conversely, bacteria and viruses can induce pyroptosis by activating caspase-4/5/11 in an NLRP3 inflammasome-independent manner. The released inflammatory cytokines not only compromise the integrity of the BRB but also exacerbate inflammation by enhancing the production of inflammatory cytokines from pathogenic T cells and promoting immune cell infiltration. MCC950 and CY-09 are inhibitors of NLRP3; necrosulfonamide is an inhibitor of gasdermin D; anakinra and canakinumab are inhibitors of IL-1β.

Clinical studies have demonstrated elevated IL-1β levels in the aqueous humor of uveitis patients, showing a positive correlation with disease activity (89, 90). Infections such as *Mycobacterium tuberculosis* or herpes simplex virus (HSV) can trigger pyroptosis by activating caspase-4, -5, and -11 to aggravate uveitis (91, 92). Pathogen-associated molecular patterns (PAMPs) or DAMPs can activate the NLRP3/ASC/caspase-1 signaling pathway, leading to pyroptosis and further disruption of the BRB (93, 94). Autoantigens, such as retinal S antigens, may mimic PAMPs and promote pyroptosis in immune cells. Upon cleavage by caspase-1, -4, -5, and -11 in retinal pigment epithelial (RPE) cells or immune cells, the pyroptosis executor protein gasdermin D forms membrane pores, leading to the release of pro-inflammatory factors such as IL-1β and IL-18, which further recruit inflammatory cells and thereby drive the inflammatory cascades in uveitis (94). Notably, IL-1β amplifies Th17 cell (derived from CD4^+^ T cells under the induction of TGF-β, IL-6, and IL-21) responses, aggravating the progression of autoimmune uveitis such as Vogt-Koyanagi-Harada (VKH) syndrome (see **Figure 1**) (95).

MCC950, a selective NLRP3 inhibitor, has been shown to reduce IL-1β secretion and attenuate retinal inflammation in the experimental autoimmune uveitis (EAU) model (53). CY-09, a specifical inhibitor of NLRP3 ATPase activity, alleviates uveitis by directly binding to the ATP-binding site within the NACHT domain of NLRP3, thereby blocking ATP binding to NLRP3 (54). Colchicine exerts anti-inflammatory effects in Behçet’s disease by inhibiting NLRP3 inflammasome assembly. However, its efficacy is relatively modest, being best suited for recurrent oral ulcers or mild arthritis, particularly in the absence of significant ocular inflammation. Accordingly, it serves as an adjunctive therapy for uveitis and plays a role in long-term maintenance and relapse prevention (96). Necrosulfonamide, an inhibitor of gasdermin D pore formation, attenuates the release of pro-inflammatory cytokines such as IL-1β and IL-18; however, its ocular delivery method requires further optimization (55). Additionally, anti-IL-1 therapies such as anakinra and canakinumab are effective for refractory uveitis, though further clinical validation is necessary (56–58). Baicalin, a flavonoid compound derived from the chinese herbal medicine scutellaria baicalensis, exhibits antioxidant and anti-inflammatory properties and has been shown to inhibit uveitis via suppression of the NLRP3/caspase-1 signaling pathway (97–99). Nanocarrier-mediated delivery of gasdermin D siRNA represents a promising strategy for uveitis treatment (100, 101).

These findings demonstrate that pyroptosis exacerbates uveitis-associated inflammatory damage primarily via the NLRP3/gasdermin D axis. Targeting this pathway, via NLRP3 inhibitors, gasdermin D-targeting agents, or cytokine blockade, holds significant therapeutic promise. However, challenges such as ocular drug delivery barriers and long-term safety must be resolved. Future studies should prioritize clinical translation and the development of optimized combination therapies.

## 3 Ferroptosis

The concept of ferroptosis was first proposed by the Brent R. Stockwell laboratory in 2012 as a regulated form of cell death triggered by the excessive accumulation of iron-dependent lipid ROS (102). Its discovery stemmed from studies on the mechanism of cancer cell death induced by the small-molecule compound erastin, which is therefore recognized as the first identified specific inducer of ferroptosis and a key tool for studying its underlying mechanisms. Ferroptosis does not rely on caspase activation and can be inhibited by iron chelators or antioxidants, thereby distinguishing from apoptosis, necrosis, or pyroptosis. The core features of ferroptosis include glutathione depletion, lipid peroxidation, and mitochondrial morphological changes such as shrinkage and loss of cristae (39, 40).

**Figure 2:**
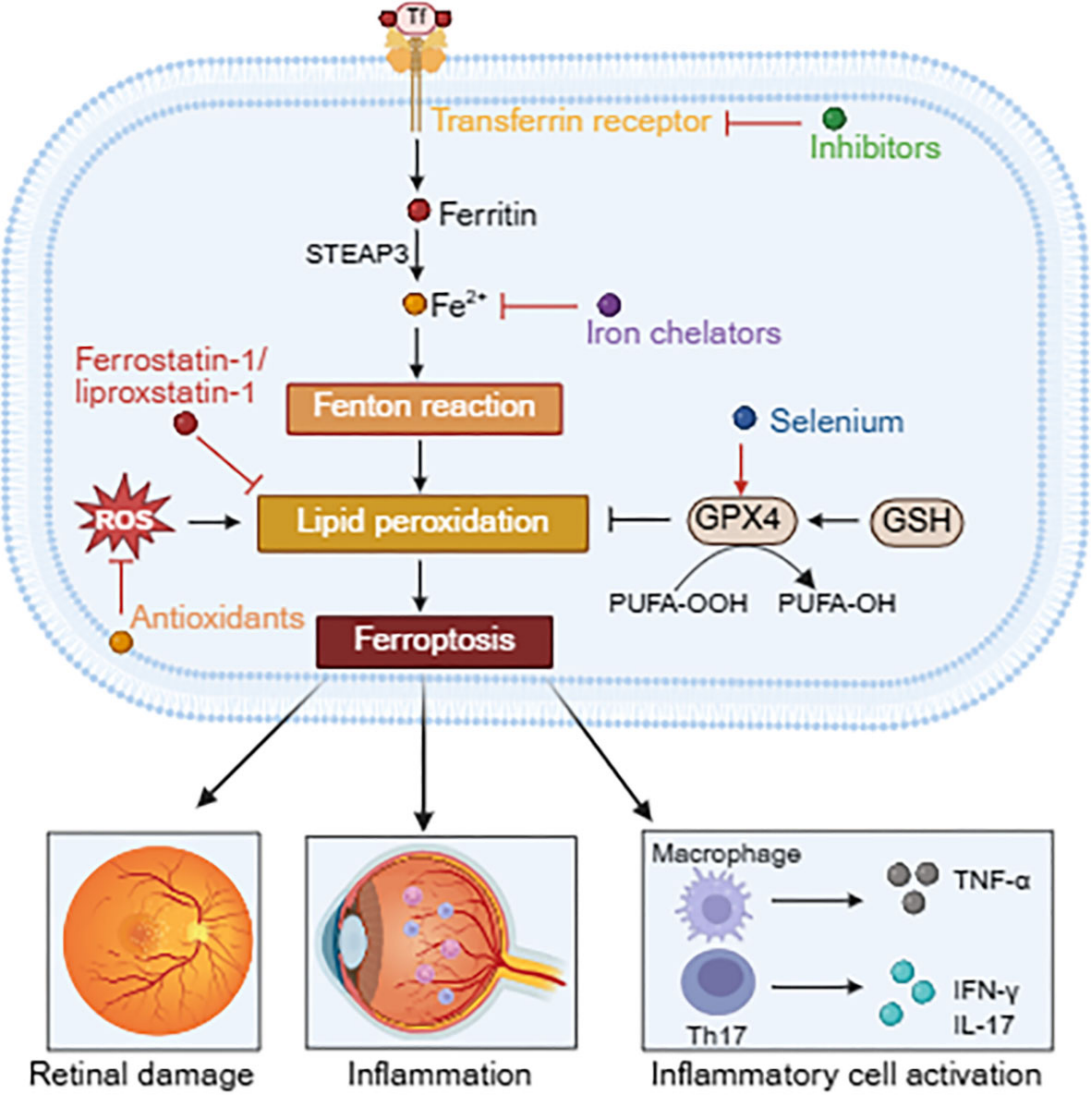
The pro-inflammatory mechanisms of ferroptosis in uveitis and targeted drugs. Excessive iron accumulation promotes the Fenton reaction, generating hydroxyl radicals and lipid peroxidation, thereby facilitating ferroptosis. DAMPs released from ferroptosis promote uveitis progression by modulating retinal cell damage and inflammation, while also activating macrophages and Th17 cells to produce pro-inflammatory cytokines, forming a “ferroptosis-inflammation” positive feedback loop. Therapeutic agents include iron uptake inhibitors, iron chelators, ferrostatin-1 or liproxstatin-1 (which inhibit lipid peroxidation), antioxidants, and selenium supplements.

Recent studies have demonstrated that ferroptosis is associated with various inflammatory and degenerative diseases, including uveitis (103–106). In uveitis patients, excessive iron accumulation in the aqueous humor or retinal tissue promotes the Fenton reaction, leading to hydroxyl radical generation and exacerbating oxidative stress-induced tissue damage (107). Moreover, ferroptosis contributes to uveitis progression by regulating inflammatory responses and retinal cell damage, including ROS overproduction, which induces lipid peroxidation in RPE and photoreceptor cells, ultimately leading to cell death and aggravated inflammation (108). In addition, DAMPs released during ferroptosis such as high mobility group box 1 (HMGB1) activate macrophages and Th17 cells, promoting the secretion of pro-inflammatory cytokines such as TNF-α, IFN-γ and IL-17, thereby establishing a ‘ferroptosis-inflammation’ positive feedback loop (see **Figure 2**) (33, 109).

Targeting iron metabolism pathways may offer novel therapeutic strategies for refractory uveitis, such as through inhibiting TfR1 or DMT1 to block iron uptake (59, 60). Iron chelators such as deferoxamine and deferasirox suppress lipid peroxidation by sequestering intracellular free iron (61–64). However, systemic administration of these agents carries the risk of anemia, necessitating the development of localized ocular delivery systems such as nanoparticles. Ferroptosis inhibitors such as ferrostatin-1 or liproxstatin-1 directly scavenge lipid radicals and preserve GPX4 activity (65–67). Although selenium supplementation has been shown to upregulate GPX4 expression, its therapeutic efficacy in uveitis must be confirmed through clinical trials (68, 69).

Ferroptosis inhibitors, when combined with conventional immunosuppressants, may exert synergistic effects in reducing both inflammation and cellular damage. However, current studies on ferroptosis inhibitors remain largely confined to preclinical animal models. Further studies are required to optimize their intraocular permeability and evaluate long-term safety profiles, as well as to elucidate the cell-type-specific roles of ferroptosis in the pathogenesis of uveitis.

## 4 Apoptosis

In 1972, Kerr, Wyllie, and Currie proposed a programmed cell death mechanism distinct from necrosis, termed apoptosis (110). The term ‘apoptosis’ originates from the Greek word for ‘shedding’, akin to leaves falling from a tree. It is an active and orderly cellular process, morphologically characterized by cell shrinkage, chromatin condensation, formation of membran-bound apoptotic bodies through cell membrane blebbing, and subsequent phagocytosis by neighboring cells (non-professional phagocytes: comprising epithelial cells, endothelial cells, mesenchymal cells, and tissue-resident cells) or macrophages (42, 43). In contrast to pyroptosis, which is accompanied by a robust inflammatory response, apoptosis is typically an immunologically silent process (111). Apoptosis is primarily initiated via two core pathways that converge at a common execution phase. The extrinsic pathway is triggered by the binding of death ligands, including FasL and TNF-α, to their cognate death receptors, leading to recruitment of the initiator caspase-8 (44). In parallel, the intrinsic pathway senses mitochondrial damage and recruits the initiator caspase-9. Both pathways ultimately activate the downstream executioner caspases, caspase-3 and caspase-7, which orchestrate the coordinated dismantling of the cell (45).

**Figure 3:**
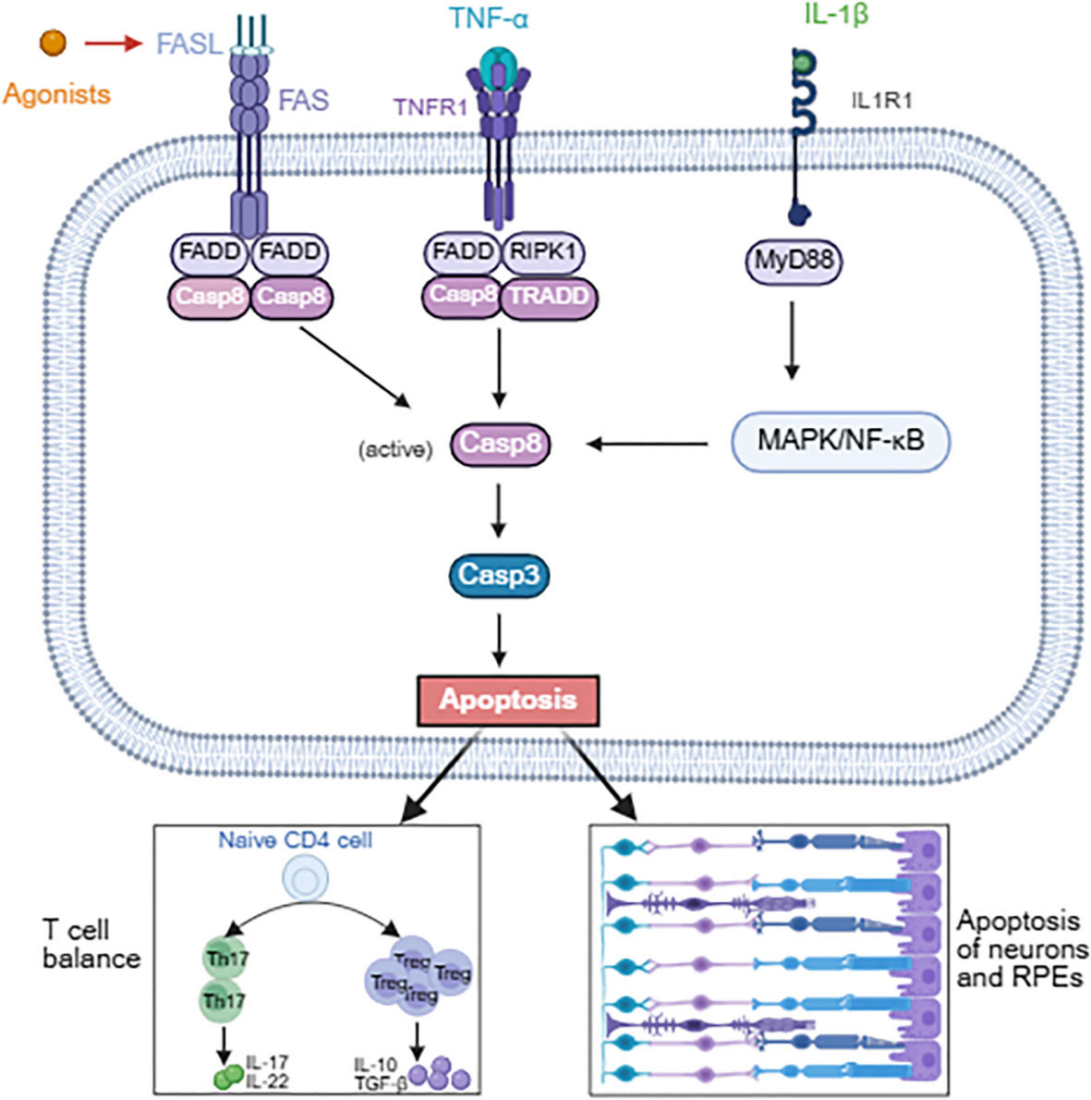
The role of apoptosis in the pathogenesis of uveitis and targeted therapeutic strategy. The Fas/FasL and TNF-α/TNFR1 pathways assemble into complexes with FADD and caspase-8, driving caspase-8 activation. IL-1β engages IL-1RI to recruit MyD88, activating the MAPK/NF-κB axis, which in turn indirectly promotes caspase-8 activation. Activated caspase-8 induces apoptosis by triggering the effector caspase, caspase-3. Apoptosis serves a dual function: it maintains T cell homeostasis to avert inflammation, yet concurrently promotes neuronal and RPE cell death. Recombinant Fas/FasL agonists offer a promising therapeutic approach for uveitis.

Dysregulation of apoptosis contributes to the pathogenesis of uveitis. During immune tolerance induction in uveitis, activation of the Fas/FasL pathway has been shown to occur concomitantly with an increase in regulatory T cell (Treg) numbers (73, 74). In contrast, Fas/FasL deficiency impairs downstream caspase-8 activation, resulting in reduced Treg numbers and the onset of uveitis. Pro-inflammatory cytokine TNF-α engages tumor necrosis factor receptor 1 (TNFR1) to promote the assembly of a multi-protein complex composed of RIPK1, TNF receptor associated death domain (TRADD), Fas associated death domain (FADD), and caspase-8. This complex triggers caspase-8 activation, which in turn induces apoptosis of retinal neurons, ultimately contributing to visual impairment (112, 113). IL-1β initiates signaling by forming a receptor complex with IL-1 receptor I and its accessory protein IL-1RAcP, leading to recruitment of the adaptor protein MyD88 and subsequent activation of downstream MAPK and NF-κB pathways. These signaling ascades indirectly induce apoptosis of retinal pigment epithelial cells (see **Figure 3**) (114).

The precise regulation of apoptosis offers a novel therapeutic strategy for uveitis, however further clinical evaluation is necessary to confirm its safety and efficacy. Local application of recombinant FasL or Fas agonists is a potential strategy for uveitis treatment (72). Apoptosis modulators may be combined with biologic agents, such as the anti-TNF-α drug adalimumab, to enhance uveitis treatment efficacy.

## 5 NETosis

In 2004, Brinkmann et al. observed that activated neutrophils release chromatin fibers to trap and eliminate pathogens, thereby proposing the concept of NETosis, a unique form of programmed cell death (115). These reticular structures primarily consist of DNA, histones, and granular proteins (46, 47). Subsequent studies revealed that NETosis is not only involved in host defense against infections but also contributed to the pathogenesis of autoimmune diseases and inflammatory disorders such as uveitis (116–120).

**Figure 4:**
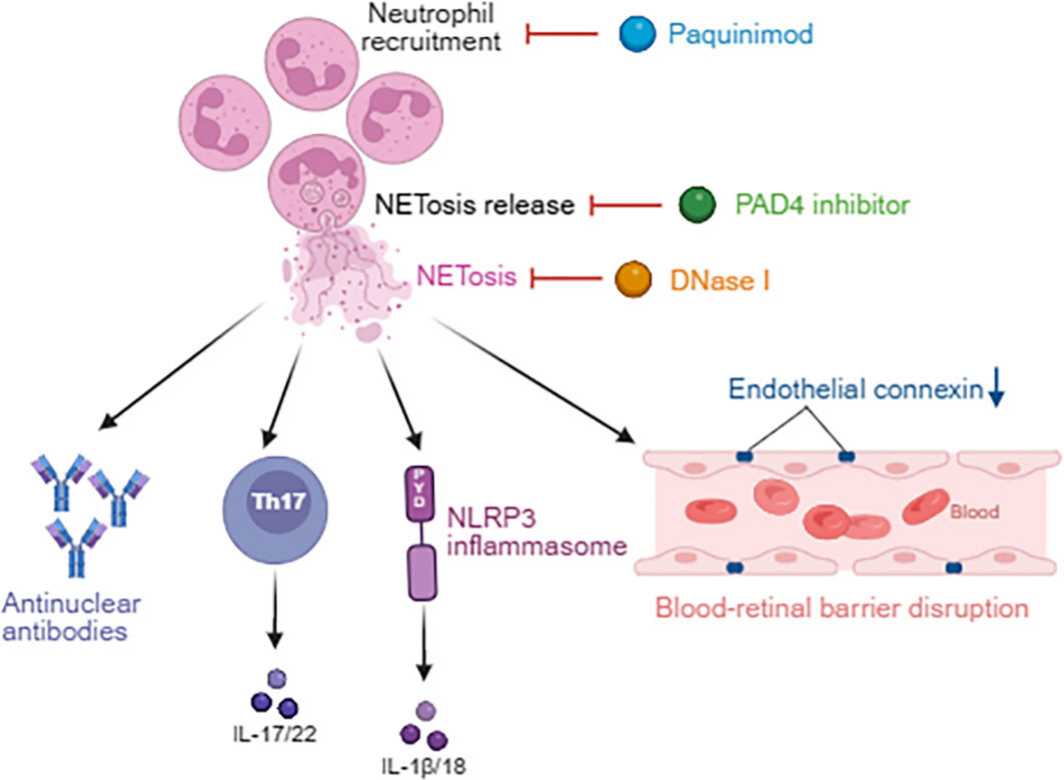
The pro-inflammatory mechanisms of NETosis in uveitis and targeted therapies. NETs harbor autoantigens that trigger anti-nuclear antibody production and directly activate both Th17 cells and the NLRP3 inflammasome. Furthermore, NET-derived elastase compromises the integrity of BRB. Therapeutic interventions such as paquinimod (which suppresses neutrophil recruitment), PAD4 (which blocks NET release), and DNase I (which cleaves NETs) all demonstrate marked efficacy in uveitis treatment.

Study has shown that NETs can directly activate Th17 cells, thereby exacerbating EAU inflammation (34, 81). Additionally, the DNA and histones within NETs may act as autoantigens, triggering the production of antinuclear antibodies and contributing to the development of autoimmune uveitis, such as VKH syndrome and Behçet’s disease (121). Furthermore, HMGB1 and cathelicidin antimicrobial peptide (LL-37) in NETs can activate the NLRP3 inflammasome, thereby promoting IL-1β release and worsening uveitis (122–125). Finally, neutrophil elastase in NETs degrades endothelial junction proteins such as vascular endothelial cadherin, leading to BRB disruption (see **Figure 4**) (126).

S100A8/A9, members of the S100 calcium-binding protein family predominantly expressed in neutrophils, exist as a stable heterodimer and exert their biological functions primarily within the intracellular compartment. Upon inflammatory stimulation, they are released into the extracellular milieu via non-classical secretory pathways, where they mediate neutrophil recruitment to sites of inflammation and modulate neutrophil adhesion and migration. S100A8/A9 inhibitors such as paquinimod can reduce neutrophil recruitment and inhibit NETosis formation (75, 76). PAD4 belongs to the peptidylarginine deiminase family and catalyzes the citrullination of arginine residues on histones. This post-translational modification reduces the electrostatic affinity between histones and DNA, thereby promoting chromatin decondensation and subsequent release. PAD4 inhibitors suppress NETosis release, demonstrating therapeutic potential in animal models of uveitis (77, 78). DNase I, which degrades NET-associated DNA, has been tested in clinical trials for systemic lupus erythematosus and may also be applicable to uveitis treatment (79, 80). Additionally, anti-TNF-α agents such as adalimumab have shown efficacy in Behçet’s disease, possibly in part by reducing NET formation (127, 128).

## 6 Autophagic cell death

Christian de Duve observed in 1963 that cells encapsulated intracellular components by forming double-membrane structures and delivered them to lysosomal degradation under electron microscopy, leading to the proposal of the concept of autophagy (129). Subsequent studies using yeast models identified ATGs, including ATG1, ATG3, ATG7, ATG17, ATG19, ATG23, ATG24, ATG29, and ATG32, and elucidated the underlying molecular mechanisms (130, 131).

In essence, autophagy is defined as a cellular process in which cells form double-membrane vesicles, named autophagosomes, to sequester damaged organelles, protein aggregates, or intracellular pathogens under stress conditions. These vesicles subsequently fuse with lysosomes, where the enclosed contents are degraded into amino acids, fatty acids, and other reusable substances, thereby maintaining intracellular homeostasis (132, 133). Based on the substrate delivery mode, autophagy is classified into three types: macroautophagy, microautophagy, and chaperone-mediated autophagy (134–136). Morphologically, autophagy is characterized by the formation of autophagolysosomes and can be divided into six sequential stages: initiation, nucleation, elongation, maturation, fusion, and degradation, all of which rely on the coordinated action of ATG family proteins (50, 137, 138).

Autophagy is involved in the pathogenesis of uveitis by regulating immune responses and cell survival. In infectious uveitis, autophagy can promote the clearance of intracellular pathogens; however, certain pathogens, such as HSV type 1, may evade immune detection by inhibiting autophagy (139–141). Additionally, autophagy helps the removal of damaged mitochondria, suppresses NLRP3 inflammasome activation and subsequent inflammatory cytokine production by reducing ROS release, and promotes the survival of RPE and photoreceptor cells, thereby mitigating inflammatory damage (142–144). Moreover, study has shown that impaired autophagy leads to excessive activation of Th17 cells, thereby exacerbating EAU inflammation (145).

The mTOR inhibitor rapamycin has been shown to activate autophagy and thereby reduce inflammation in the EAU model, whereas autophagy inhibitors such as chloroquine and hydroxychloroquine block autophagosome-lysosome fusion, offering potential therapeutic benefits in cases of infectious uveitis driven by excessive autophagy (82, 83). Combining mTOR inhibitor (rapamycin) with anti-inflammatory drug (dexamethasone), may enhance therapeutic efficacy by synergistically suppressing EAU. However, strategies targeting ATGs or microRNAs remain largely experimental and require further investigation (146–149). In conclusion, autophagy exhibits dual roles- both anti-inflammatory and pro-inflammatory-requiring precise regulation based on uveitis subtypes for targeted therapy.

## 7 Necroptosis

The discovery and conceptualization of necroptosis represent a significant milestone in the field of cell death research. It overturned the traditional view that necrosis is an unregulated accidental event, demonstrating that necrosis can also occur as a precisely controlled programmed process. In 2005, a research team led by Junying Yuan at Harvard Medical School identified and reported a small molecule compound called necrostatin-1, which efficiently and specifically inhibits TNF-induced regulated necrosis (150). Based on this discovery, they formally named this regulated necrotic process “necroptosis.”

Necroptosis is a caspase-independent form of programmed necrosis, activated by death receptors (such as TNFR and Fas) or pattern recognition receptors (such as TLRs). It is mediated by the RIPK1/RIPK3/MLKL signaling axis, ultimately leading to plasma membrane rupture, release of cellular contents, and inflammatory responses. Distinct from apoptosis, necroptosis exhibits characteristic necrotic morphology: including cell swelling, plasma membrane rupture, organelle swelling and dysfunction, and the formation of RIP1-RIP3 necrosomes (151). Necroptosis often coexists with apoptosis and pyroptosis in the same pathological process, a phenomenon termed PANoptosis (152).

**Figure 5:**
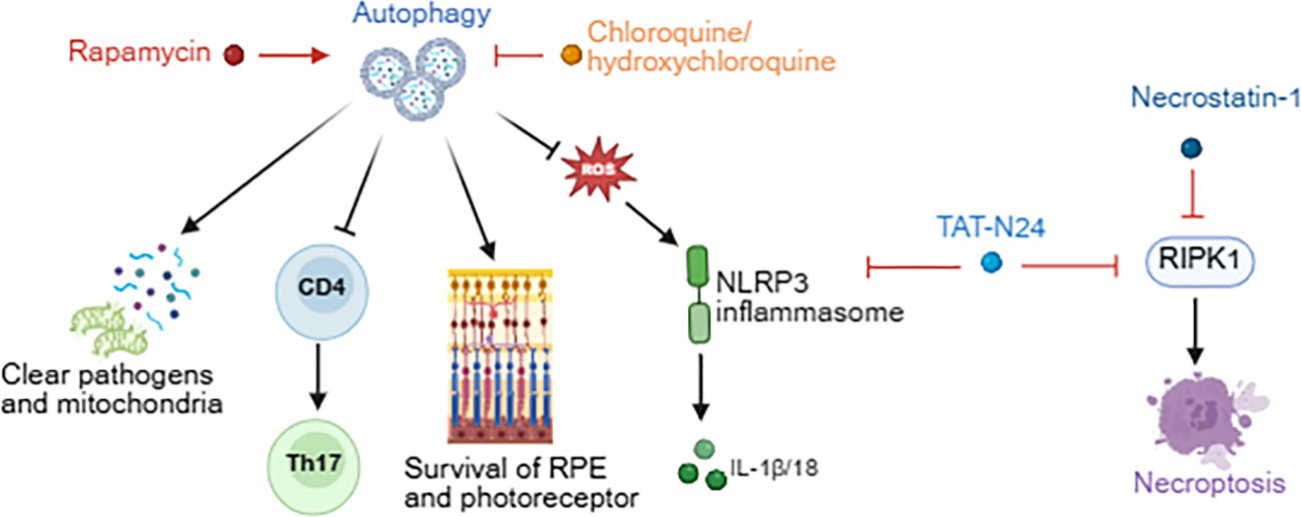
Role of autophagy and necroptosis in uveitis and targeted therapies. Autophagy exerts anti-inflammatory effects in uveitis by clearing pathogens and damaged mitochondria, sustaining retinal cell survival, and suppressing both Th17 cell differentiation and NLRP3 inflammasome activation. The mTOR inhibitor rapamycin, which activates autophagy, as well as chloroquine and hydroxychloroquine, which inhibit excessive autophagy, have each demonstrated therapeutic benefits in uveitis. Furthermore, TAT-N24 and necrostatin-1 suppress necroptosis by inhibiting RIPK1, thereby alleviating the associated inflammatory response.

Research on the mechanism of necroptosis in uveitis is still in its early stages; however, existing studies have revealed key clues suggesting that it likely plays an important role in the pathogenesis of uveitis through crosstalk with other inflammatory cell death pathways. Zhu et al. found that TAT-N24, a p55PIK inhibitor, exerts significant anti-inflammatory effects in a mouse model of uveitis by inhibiting the Z-DNA binding protein (ZBP) 1-PANoptosome complex (which includes RIPK1 and RIPK3) as well as the NLRP3 inflammasome (35). Necrostatin-1 has been shown to protect retinal ganglion cells in a retinal ischemia-reperfusion injury model, and its combination with NLRP3-targeting agents such as colchicine is particularly suitable for refractory cases, including Behçet’s disease-associated uveitis (see **Figure 5**) (84).

## 8 Conclusions and perspectives

In recent years, uveitis research has expanded beyond a primary focus on inflammatory cell infiltration to increasingly emphasize the central role of cell death in disease initiation, progression, and resolution. In summary, cell death plays a central role in the onset and progression of uveitis, involving diverse molecular mechanisms and immune regulatory networks. However, different intraocular cells, such as RPE cells and microglia, respond differentially to death signals. Single-cell RNA sequencing (scRNA-seq) can help elucidate the heterogeneity in cell death patterns among these cells during uveitis, as well as the crosstalk between different cell death pathways. Additionally, several challenges remain in uveitis research: 1) The EAU model cannot fully recapitulate the complexity of human uveitis, necessitating the development of more clinically relevant models; 2) Long-term suppression of specific cell death pathways may compromise host defense or tissue homeostasis; 3) Different uveitis subtypes may require distinct cell death intervention strategies.

Different types of cell death pathways exhibit distinct biomarkers in uveitis: pyroptosis is characterized by the NLRP3 inflammasome and gasdermin D; ferroptosis is associated with GPX4 dysregulation and ROS accumulation; apoptosis is marked by FasL engagement and caspase-3 activation; NETosis is defined by PAD4 activation and the presence of MPO-DNA complexes; autophagic cell death involves autophagy-related proteins such as Beclin-1 and LC3; and necroptosis is centered on phosphorylated RIPK1/3 or MLKL. Given that multiple forms of cell death often coexist in uveitis, the development of combined biomarker panels-such as those assessing “pyroptosis and necroptosis” or “ferroptosis and NETosis”-may enable more precise characterization of disease subtypes and activity states.

**Figure 6:**
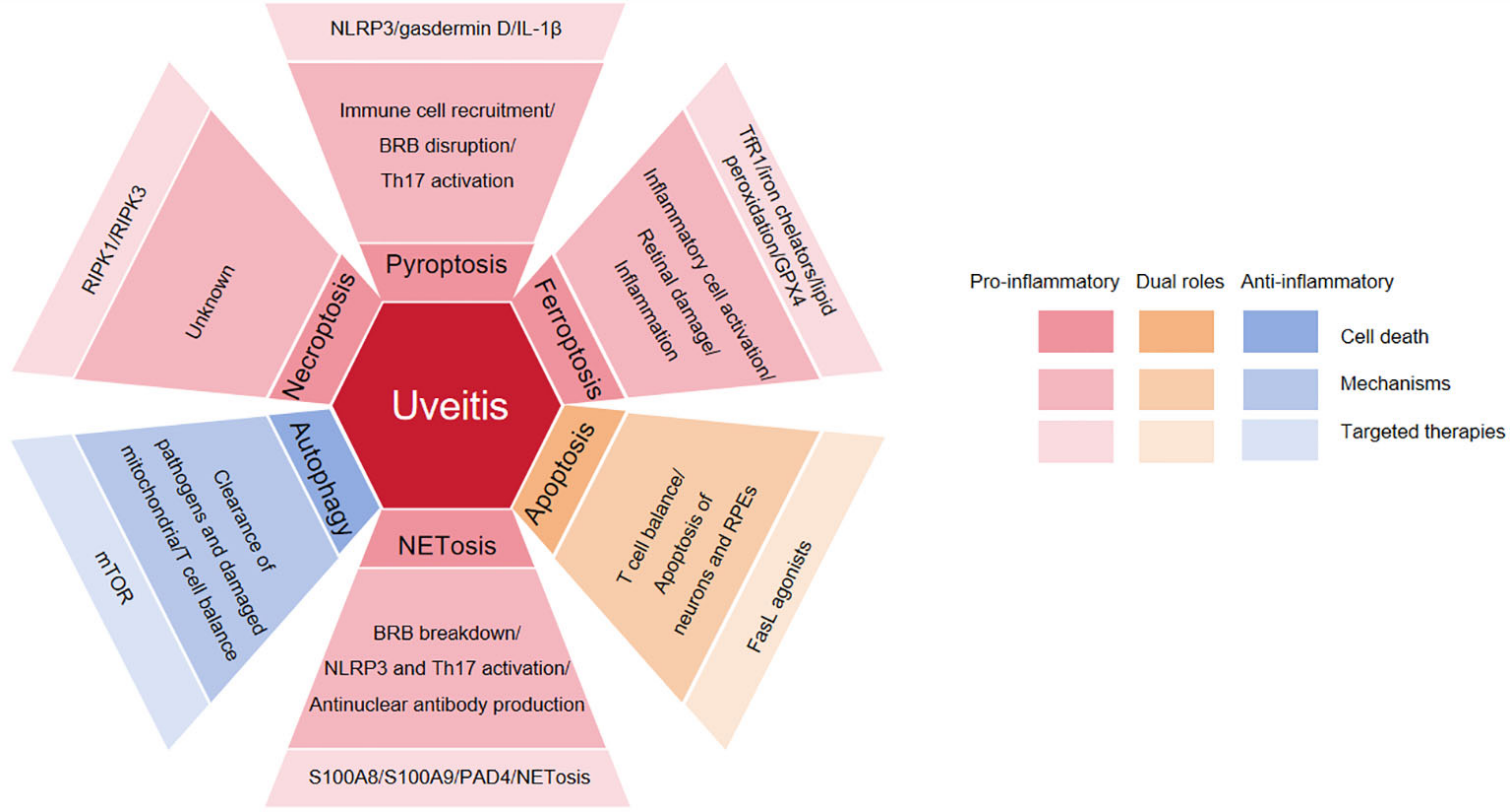
The role, mechanisms, and therapeutic targeting of diverse cell death pathways in uveitis. Pyroptosis, ferroptosis, NETosis, and necroptosis contribute to the pathogenesis of uveitis, and the use of targeted inhibitors has shown therapeutic promise in managing the disease. Apoptosis plays a dual role in uveitis; targeted agonists exert therapeutic effects by modulating T cell homeostasis. Autophagy exerts anti-inflammatory effects in uveitis, highlighting the therapeutic potential of targeted activators in the treatment of this condition.

Regarding targeted therapeutic strategies, pyroptosis can be targeted with NLRP3 inhibitors (e.g., MCC950) or IL-1 receptor antagonists; ferroptosis can be addressed using ferrostatin-1 or iron chelators; during the inflammatory resolution phase, apoptosis can be promoted via FasL agonists; NETosis can be inhibited by PAD4 inhibitors or DNase I; autophagic cell death can be modulated with autophagy inhibitors such as chloroquine; and necroptosis can be targeted by inhibiting RIPK1, for instance with necrostatin-1 (see **Figure 6**). An ideal therapeutic approach should aim to inhibit pyroptosis or necroptosis during the acute inflammatory phase, while restoring apoptosis or autophagy during the resolution phase to eliminate pathogenic cells and promote tissue repair. In this context, achieving precision subtyping and individualized targeted therapy based on cell death marker profiles in patients’ intraocular fluid or peripheral blood represents a critical direction for the future of precision medicine in uveitis.

Future uveitis management may incorporate aqueous humor or serum biomarkers, such as HMGB1, lactate dehydrogenase, and IL-18, for subtype classification and prognosis prediction. Emerging therapies, including targeted drug delivery and gene editing, hold promise for clinical translation. While multi-target approaches dominate current treatment paradigms, optimizing the risk-benefit ratio remains essential.
